# Development and validation of a quality of healthy work environment instrument for shift nurses

**DOI:** 10.1186/s12912-023-01672-4

**Published:** 2024-01-12

**Authors:** Sun-Hwa Shin, Eun-Hye Lee

**Affiliations:** College of Nursing, Sahmyook University, 815, Hwarang-ro, Nowon-gu, 01795 Seoul, Republic of Korea

**Keywords:** Nurses, Work, Environment, Factor analysis, Validation study

## Abstract

**Background:**

As the importance of a healthy work environment for nurses’ good practice and patient safety has been recognized, there is a need to assess nurses’ perceptions of the quality of a healthy work environment.

**Methods:**

A conceptual framework and construct components were extracted through a literature review and in-depth interviews with shift nurses. The initial items of the instrument were developed according to the conceptual attributes, and the items were selected through content validity by ten experts. Two hundred and forty-seven shift nurses participated in this study through face-to-face surveys to test the reliability and validity of the instrument. The evaluation was used for item and confirmatory factor analyses to assess the criterion-related validity and internal consistency of the instrument. Test-retest reliability was analyzed using data from thirty-two nurses.

**Results:**

The final instrument consisted of 23 items with five components identified through confirmatory factor analysis. Criterion-related validity was established using the K-PES-NWI (r = .54). Cronbach’s alpha for the total items was 0.85, and test-retest reliability was 0.73.

**Conclusion:**

The quality of the healthy work environment instrument developed in this study was considered reliable and valid. The instrument developed in this study can be used to measure the quality of a healthy work environment as perceived by shift nurses and to identify preventive measures needed to improve the quality of the work environment.

**Supplementary Information:**

The online version contains supplementary material available at 10.1186/s12912-023-01672-4.

## Introduction

Nursing shortage is a global concern, and new graduate nurses are seen as the most important resource for addressing this shortage [1]. In a 2020 survey of resignations, the overall average resignation rate was 14.2%, and nurses with less than one year of experience had the highest resignation rate of 34.1% [2]. Poor working conditions, such as the nature of shift work, heavy workload, and poor treatment, have been reported to cause turnover among Korean nurses [3] by increasing nurses’ burnout and adversely affecting patient outcomes [4]. On the other hand, a healthy nursing work environment increases nurses’ job satisfaction and performance and promotes high quality nursing care [5, 6].

There is an increasing interest in creating a desirable nursing work environment due to its importance for patient safety and quality of care. The term “nursing work environment” not only refers to the place of work, but also includes the physical, psychological, and structural environments, relationships with coworkers, and hospital policies as well as nurses’ perceptions of them [7]. Nurses’ practices for patients in a safe environment was directly related to the quality of health care [8]. Additionally, a healthy work environment has been empirically associated with patient satisfaction, reduced turnover, increased job favorability and satisfaction, and reduced job stress and burnout [4, 5]. Turnover intention among shift nurses in Korea have been analyzed as a result of work conflicts and stressful work environment. In the United States, the Institute of Medicine (IOM) has noted that nurses’ work environment plays a crucial role in providing safe and quality patient care and has strongly recommended improving it [9]. It affects both nurses’ and patients’ performance [10], and plays a major role in nurses’ retention and health [11]. Therefore, in addition to improving the nursing work environment, we need to pay attention to improving quality.

We reviewed previous studies that assessed the work environments of nurses in Korea. One study used the Korean version of the Practice Environment Scale of the Nursing Work Index (PES-NWI), which was developed for international nurses [12]. There were also instrument that was developed and measured in the Korean cultural context [13]. In other studies, instruments have been developed and used to identify the working environment of nurses in specialized areas, such as clinical nurses and intensive care unit nurses, or to identify the organizational culture of nursing [14–16]. Most nursing work environment measurement instruments focus on the healthcare organization’s systems, organizational culture, leadership of nursing managers, and adequacy of staffing [14–17]. Most of these instruments were developed for nurse managers, experienced nurses, and nurses in specialty units, and none considered the work environment of shift nurses or examined the clinical adjustment of new nurses. They also focused on social relationships and communication, nurse manager leadership, and organizational culture but did not address the environmental factors that promote or threaten health in the physical and psychological domains that nurses experience during their work. The World Health Organization (WHO) defines health as “a state of physical, psychological, and social integrity and complete freedom from disease and infirmity.“ [18]. Therefore, there is a need to develop a tool to assess the quality of a healthy work environment for nurses by focusing on the factors that promote or threaten it.

The WHO has proposed the following dimensions of health-related quality of life: physical domain, psychological domain, level of independence, social domain, environmental domain, and spiritual domain [19]. The quality of life scales proposed by the WHO are mainly used to evaluate the effectiveness of healthcare, including health status, lifestyle, satisfaction, and well-being [20]. Quality of life refers to an individual’s perceived degree of well-being in life in the context of the culture and value systems in which they live and in relation to their goals, expectations, standards and concerns [21]. In 2001, the American Association of Critical-Care Nurses (AACN) focused on promoting a work environment that fosters excellence in patient care for nurses who provided acute and critical care. In 2005, AACN published the AACN Standards for establishing and maintaining a healthy work environment (HWE). The AACN standards propose six essential elements of a HWE: skilled communication, true collaboration, effective decision-making, meaningful recognition, appropriate staffing, and authentic leadership [17]. Therefore, it is appropriate to evaluate how nurses perceive their work environment as healthy. In this study, the WHO’s health-related quality of life domains and the AACN’s proposed healthy work environment standards were used as a conceptual framework to develop a questionnaire to measure the quality of healthy work environment.

This study aimed to develop an instrument to measure the quality of healthy work environment for shift nurses with the following specific goals: developing an instrument and evaluating the reliability and validity of the developed instrument.

## Methods

### Study design

**Fig. 1:**
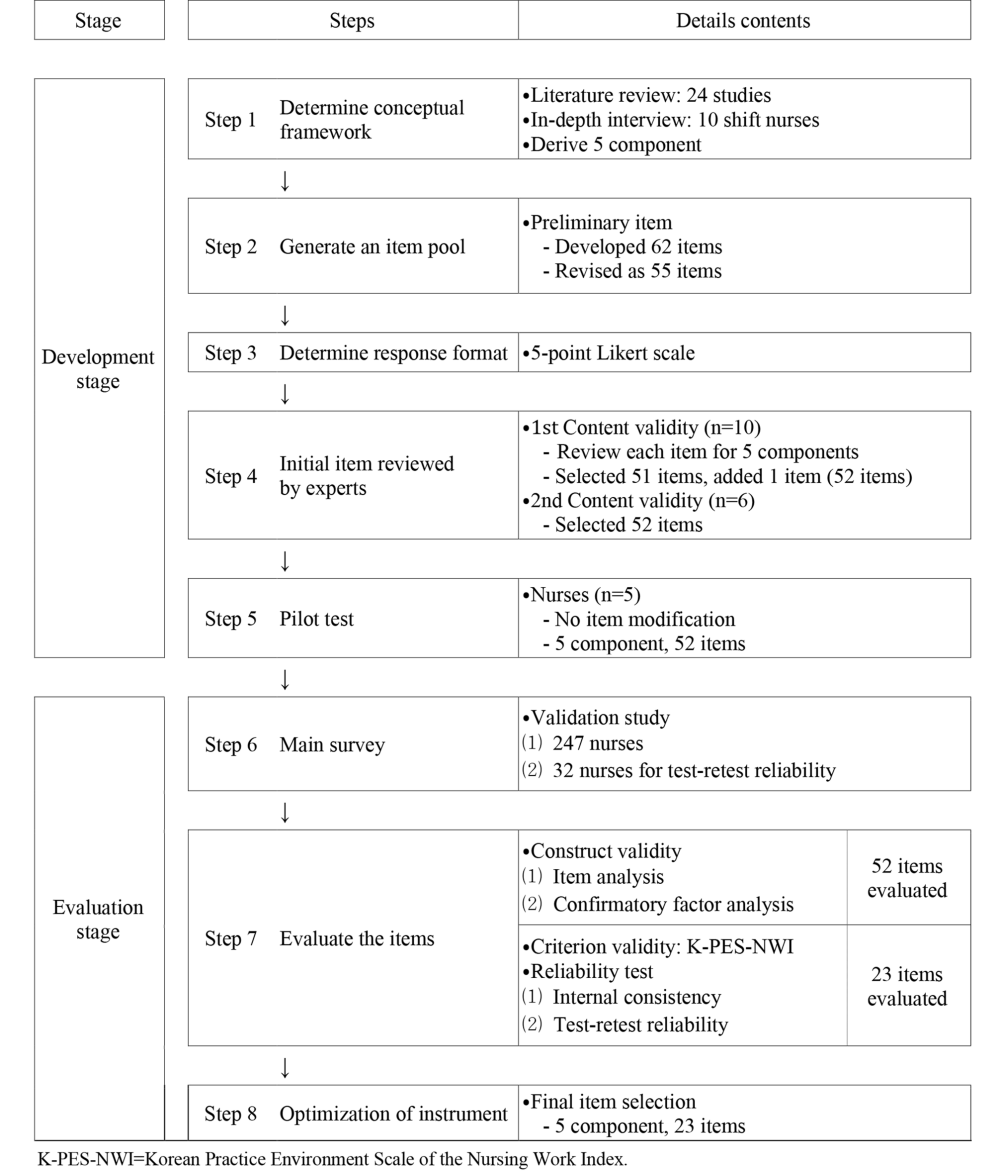
Steps of quality of healthy work environment instrument development

This study was a methodological study that develops a quality of healthy work environment instrument for shift nurses and estimates its reliability and validity. Based on the instrument development procedure presented by DeVellis [22], this study involved the stages of instrument development and evaluation (Fig. 1).

## Research procedure

### Development stage

#### Determine conceptual framework

Conducted a literature review on WHO’s health-related quality of life, work environment measurement tools, and standards for healthy work environments related to domestic and international nursing. The WHO has defined quality of life as “an individual’s perception of the place of his or her life in the context of the culture and value system in which he or she lives, in relation to his or her goals, expectations, standards, and interests [21].” Using the WHO’s health-related quality of life scale (WHOQOL), we examined the domains and facets related to the work environment. Accordingly, we identified “comfort and relaxation” and “energy and fatigue” in the physical domain, “positive emotions” and “self-esteem” in the psychological domain, “work capacity” in the independence level, “interpersonal relationships” and “social support” in the social domain, and “physical safety”, “financial support”, and “Opportunities to get involved” in the environmental domain. The conceptual framework of a healthy nursing work environment proposed by the American Association of Critical Nurses (AACN) consists of six standards: skilled communication, true collaboration, effective decision-making, appropriate staffing, meaningful recognition, and authentic leadership [23]. An analysis of instruments to measure expatriate nurse’s work environment [7, 24, 25] found that factors associated with a healthy work environment for nurses include “adequate nursing staffing”, “relationships with coworkers at work”, “leadership from managers”, “autonomy in job functions”, and “training and support for work”. An analysis of instruments developed for nurses in Korea [12, 13, 15] found that common factors related to a healthy work environment include “authentic leadership from managers”, “appropriate staffing”, “adequacy of manpower and resources”, and “interpersonal relationships, including organizational culture”. The nurses’ professional quality of life (ProQOL) instrument measured the concepts of “empathy satisfaction”, “empathy fatigue”, and “burnout” [26].

Based on the review of previous studies, this study organized the conceptual attributes of quality of healthy work environment into five components as follows: physical gratification, psychological stability, independent competency, collaborative relationship, and structural support. The attributes of each concept were organized into the indicators, with a focus on health. Physical gratification is a factor related to the physical needs of nurses experienced due to shift work and can be viewed as basic physiologic needs, such as comfort, rest, energy, fatigue, holiday coverage, and meals [27, 28]. Indicators of psychological well-being were viewed as positive self-esteem, rewards, achievements, and sense of belonging as factors related to nurses’ positive experiences [29, 30]. Indicators of independent competence were work independence, scope of work, and growth experience as factors related to nurses’ capability to do the work given to them during their shifts [31]. Indicators of social relationships included interpersonal relationships, social advocacy, true collaboration, and authentic leadership as factors related to the network of social relationships with coworkers [32, 33]. Indicators of structural support included safety and protection, positive workforce environment, financial support, and administrative support as systemic factors that support nurses’ work [34].

In addition, in-depth interviews were conducted with ten shift nurses who had been working for two years. To extract the questions for this instrument, we conducted one-on-one in-depth interviews with shift nurses. We began by asking them about the kind of work environment that they think is conducive to healthy work while working in the nursing field. Then, we asked them a series of questions related to each factor of the conceptual framework of this study. These questions were designed to explore the healthy nursing work environment experienced by shift nurses. The questions we asked were:

- What do you consider to be a physically fulfilling work environment?
- What do you think makes a work environment psychologically (mentally) stable?
- What do you think is a work environment that allows you to practice your work capabilities independently?
- What do you think fosters social (collaborative) relationships in the workplace?
- What do you consider to be a structured (institutional) supportive work environment?

We concluded the in-depth interviews by asking questions about factors that improve or deteriorate a healthy work environment for shift nurses. Through these interviews, we identified a healthy work environment as feeling a sense of belonging to the department and mutual recognition in the Psychological stability domain. In the Independent competency domain, the one-person full-filled role as a nurse was identified. In the Collaborative relationship domain, having a support system to ask for help during work and an atmosphere of teamwork were identified. These results from in-depth interviews were reflected in the development questions.

**Fig. 2:**
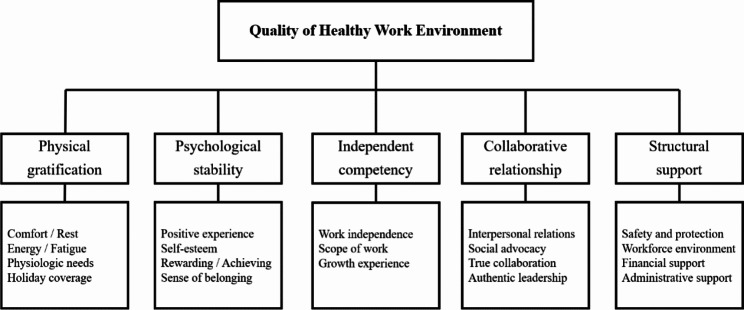
Conceptual framework for quality of healthy work environment

A conceptual framework and construct components were extracted through a literature review and interviews with shift nurses. The attributes according to the construct concept are presented in Fig. 2.

#### Generate an item pool

Preliminary items were developed for each of the five components (physical gratification, psychological stability, independent competence, cooperative relationships, and structural support) and indicators based on the conceptual attributes. First, 62 preliminary items were developed. The researchers repeatedly reviewed these items with a psychology professor who is an expert in instrument development to remove duplicates. In addition, 55 preliminary questions were created in the second round by deleting 7 items that reflected the characteristics of the subject rather than the work environment.

#### Determine response format

The format of measurement in this study was a 5-point Likert scale. The responses to the items were determined as “strongly agree” = 5 points, “agree” = 4 points, “neutral” = 3 points, “disagree” = 2 points, and “strongly disagree” = 1 point.

#### Review of initial item by experts: content validity

Regarding the second preliminary item (55 items), content validity was evaluated by requesting advice from nursing managers and experienced nurses working in clinical settings. Content validity was assessed by 10 experts, including two nursing professors with more than 10 years of clinical experience, five current nursing administrators, and three experienced nurses with more than 10 years of experience. The experts were asked to judge the relevance of the preliminary items to the concept being measured and to rate them on a scale of 1 (not at all valid) to 4 (very valid). To select valid items, the item-content validity index (I-CVI) of the preliminary items was calculated, and items with an I-CVI of 0.80 or higher were selected. Additionally, the experts were asked to provide their opinions regarding the items that needed further revision. Two items with an I-CVI of 0.80 or less and two items that were judged to have similar meaning were removed. The wording of some items was revised to reflect the experts’ opinions, and one question was added to select 52 items for the third preliminary item. The third set of preliminary items was requested to six experts for secondary content validity. All items’ I-CVI coefficients were found to be above 0.80, and the wording of 7 items was modified to maintain 52 items.

#### Consideration of validation items: pilot test

A pilot test (cognitive interviews) was conducted to evaluate the understanding of the preliminary items. We asked five clinical nurses to check the language and understanding of the third preliminary item (52 items). The questionnaire took 4 to 5 min to complete, and the understanding of the items were rated as “easy to understand (3 points)” and “very easy to understand (4 points),” indicating that it was generally easy to understand the meaning of the items.

### Evaluation stage

#### Participants of main survey

The participants in the main survey were clinical nurses working in general hospitals with more than 300 beds located in Seoul and Gyeonggi province. The inclusion criteria were those who worked the third or second shift, understood the purpose of the study, and voluntarily agreed to participate. The exclusion criteria were those working full-time in outpatient and specialty units and those working as physician assistants (PAs). Based on the rationale that the number of participants needed for the study should be five times the number of questions to be developed [35], the goal was 250 participants.

#### Data collection period and methods

Data collection for this study was conducted through a face-to-face survey in two general hospitals, after obtaining IRB approval and permission from the nursing department. Two research assistants were selected with the help of the nursing department and preliminary training was provided to them on the purpose of the study and research procedures. The research assistants visited each ward, explained the purpose of the study and how to conduct the study, and obtained written consent from patients who expressed their willingness to participate. Hospital A collected data from August 16 to 31, 2022, from 150 nurses who participated in the survey. Hospital B collected data from September 22 to October 8, 2022, from 100 nurses who participated in the survey. Among the 250 nurses, the data of 3 nurses with less than 6 months of clinical experience were removed, and the data of a total of 247 nurses were analyzed.

### Research instruments

The instrument validation questionnaire consisted of 10 items on general characteristics, 52 items on the quality of healthy work environment (QHWE) developed in this study, and 29 items on the K-PES-NWI for validation. The K-PES-NWI was developed by Lake [7] and later adapted by Cho et al. [12] for reliability and validity in Korea. The K-PES-NWI consists of 29 items and five sub-scales (nurse participation in hospital affairs, nursing foundations for quality of care, nurse manager ability, leadership, and support of nurses, staffing and resource adequacy, collegial nurse-physician relations). Responses to the K-PES-NWI items were measured on a 4-point Likert scale, with higher scores indicating more positive perceptions of the nursing work environment. The reliability of the instrument is based on Cho et al.’s study [12], whose Cronbach’s α was 0.93, and in this study, it was 0.92.

### Evaluate the items

Based on the main survey data, the item-total correlation coefficient was calculated for all items. Item analysis and confirmatory factor analysis were conducted to evaluate construct validity. Criterion validity was confirmed by analyzing the correlation between the instrument developed in this study and K-PES-NWI. The Cronbach’s α coefficient of the developed instrument was calculated to estimate the reliability. The test-retest reliability was analyzed by surveying 32 nurses four weeks later [36, 37].

### Ethical considerations

Prior to collecting the main survey data, the Institutional Review Boards of S University (IRB No. SYU 2022-03-006) and Hospital B (IRB No. SEOUL 2022-08-009), reviewed the data. For the survey, the researcher and a research assistant visited the wards to meet with nurses working in the two general hospitals and explained the study’s purpose, procedures, and about voluntary participation. The informed consent form was presented to the participants explaining that they could stop answering the questionnaire at any time without being penalized for stopping or withdrawing from the study. In addition, the ethical aspects and confidentiality of the participants were explained, and written informed consent was obtained. All participants who completed in the survey were offered a gift certificate.

### Data analysis and statistical methods

The SPSS 25.0 and AMOS 23.0 program (IBM Corp., Armonk, NY, USA) were used for the analysis of the instrument validation phase. The general characteristics of the participants were analyzed using frequency and descriptive statistics. For item analysis, the mean, standard deviation, skewness, and kurtosis values were checked for preliminary items, and correlation coefficients between the total score and each item were calculated to remove items with correlation coefficients below 0.30 [38]. Confirmatory factor analysis was conducted to examine the structural relationships among the sub-factors according to the conceptual framework of construct validity. The maximum likelihood estimation method was used because the skewness and kurtosis values of the collected individual items assumed normality. To evaluate the fit of the model, we examined the χ^2^ value and the degrees of freedom ratio, standardized root-mean-square residual (SRMR) using residuals, and root mean square error of approximation (RMSEA). We examined the incremental fit index, Turkish-Lewis index (TLI), and the relative fit index, comparative fit index (CFI). Convergent validity and discriminant validity were checked by examining the correlation coefficient, standard error (SE), construct reliability (CR), and average variance extract (AVE) among the sub-factors [39]. Criterion validity was assessed using the K-PES-NWI. Internal consistency was analyzed using Cronbach’s ⍺ coefficient, and test-retest reliability was analyzed by Pearson correlation coefficient.

## Results

### General characteristics

The participants of 217 (87.9%) in this study were women. The average age was 27.6 years (± 3.41), with 191 (77.3%) in their 20s. The marital status of 221 participants (89.5%) was single, and the highest level of education was a four-year bachelor’s degree with 232 (93.9%). No religion accounted for 165 (66.8%), and the years of career was 96 (38.9%) for 3–5 years, 79 (32.0%) for 2 years or less, and 72 (29.1%) for 6 years or more. In terms of department, 126 (51.0%) were in internal medicine wards, 67 (27.1%) in surgical wards, and 54 (21.9%) in special wards (ER, ICU, DR, NR etc.). Health status was moderate with 113 (45.7%), and job satisfaction was moderate with 123 (49.8%). Regarding future work plans, “I want to work as long as necessary” accounted for the majority of 125 (50.6%) (Table 1).

**Table 1:** General Characteristics of the Participants (N = 247)

Characteristics	Categories	n (%)
Sex	Male	30(12.1%)
	Female	217(87.9%)
Age (year)	20∼29	191(77.3%)
	Over 30	56(22.7%)
Marital status	Single	221(89.5%)
	Married	26(10.5%)
Education	Three-year bachelor’s degree	9(3.6%)
	Four-year bachelor’s degree	232(93.9%)
	Graduate School	6(2.4%)
Religion	None	165(66.8%)
	Has	82(33.2%)
Career (year)	Less 2	79(32.0%)
	3∼5	96(38.9%)
	More 6	72(29.1%)
Department	Internal medicine wards	126(51.0%)
	Surgical wards	67(27.1%)
	Special wards (ER, ICU, DR, NR ect.)	54(21.9%
Health status	Healthy	107(43.3%)
	Moderate	113(45.7%)
	Unhealthy	27(10.9%)
Job satisfaction	Satisfied	50(20.2%)
	Moderate	123(49.8%)
	Dissatisfied	74(30.0%)
Future work plans	I want to work as long as possible	53(21.5%)
	I want to work as long as necessary	125(50.6%)
	I want to quit as soon as possible	45(18.2%)
	I’m not sure	24(9.7%)

ER = Emergency Room; ICU = Intensive Care Unit; DR = Delivery Room; NR = Nursery Room

### Items analysis

For the first evaluation, the mean, standard deviation, skewness, and kurtosis values of the 52 preliminary items were reviewed. The mean values of the items ranged from 2.01 to 4.08, with no extremes. The skewness values ranged from − 0.72 to 0.78, with an intercept of less than 3. The kurtosis values ranged from − 1.03 to 2.03, with an intercept of less than 7. All items met the normality criteria.

Internal consistency of instrument was assessed with Cronbach’s alpha coefficient and item total score correlations. Cronbach’s alpha coefficient of at least 0.60 ~ 0.80 was required and item total score correlations of at least 0.30 in each item. If the correlation coefficient between the total score and each item was less than 0.30, the items with low discriminatory power were removed. The correlation coefficient between the total score of healthy work environment and each item ranged from 0.09 to 0.59. Among these, eight items (x4, x6, x7, x14, x15, x16, x34, x37) whose item-total correlation coefficient was less than 0.30 were removed. The reliability of the 52 items was 0.91, and after removing the eight items, the reliability of the 44 items increased to 0.92.

### Evaluate construct validity

For construct validity, confirmatory factor analysis was conducted to evaluate that the items form an appropriate factor structure. The sample adequacy test was conducted to examine whether the collected data had the minimum conditions for conducting factor analysis. In this study, the KMO (Kaiser Meyer Olkin) value was 0.88, and Bartlett’s test of sphericity was also statistically significant (χ^2^ = 5878.33, *p* < .001), indicating that the data were suitable for factor analysis.

Confirmatory factor analysis is a method to confirm the hypothesis of the relationship between variables by considering the measurement error of the research model [40, 41]. This study constructed the constructs and developed the items based on the previous studies. Therefore, a confirmatory factor analysis was conducted to evaluate whether the hypotheses of the research model were supported by the actual data collected. As a result of checking the fit indices of the initial model, the model fit was inadequate with χ^2^ and the ratio of degrees of freedom being 3.41, TLI 0.59, CFI 0.62, SRMR 0.11, and RMSEA 0.10. Therefore, to improve the fit indices, the research model was modified by removing items with standardized factor coefficients of 0.40 or less for each item and removing items based on the modification indices (MI) of error correlation [39]. In particular, items with a high MI index between the errors of the measured variable and other latent variables were removed if they were likely to be considered to be highly similar to items in other sub-factors, or if the attributes of the items could change depending on the interpretation of the participants after reviewing the contents of each item. The maximum likelihood estimation method was a technique that utilizes all information, so the factor analysis was performed iteratively by removing items one by one, as modifying one item affects the entire model.

First, two items (x26, x27) with standardized factor coefficients below 0.40 and an item (x47) with a high MI index were removed. Second, items with a standardized factor coefficient of 0.40 or less (x28) and items with a high MI index (x25, x48, x59) were removed. Third, items with high MI index (x39, x50, x52, x56) were removed. By repeating this process, factor analysis was performed by removing the items with high MI index (x11, x35, x36, x40, x41, x44, x51, x53, x54, x60) one by one. As a result, 21 items were removed, resulting in the final 23 items. For each sub-factor, it consisted of 4 items for factor 1, 4 items for factor 2, 5 items for factor 3, 5 items for factor 4, and 5 items for factor 5.

The goodness of fit of the final model was 1.84 with a χ2 value and a ratio of degrees of freedom below 3, and the TLI of 0.90 and CFI of 0.91 were above the standard value of 0.90. SRMR 0.07 and RMSEA 0.06 were below the criterion value of 0.80, and the 90% confidence interval of RMSEA was 0.05 ~ 0.07, all below 0.10, indicating that the fit of the final model was very good (Table 2).

**Table 2:** Model fit indices for quality of healthy work environment (N = 247)

Model	*χ* ^2^	*df*	*p*	χ^2^/*df*	TLI	CFI	SRMR	RMSEA (90% CI)
Initial research model (5-factor correlation)	3039.23	892	< 0.001	3.41	0.59	0.62	0.11	0.10 (0.09∼0.10)
Modified research model (5-factor correlation)	403.53	219	< 0.001	1.84	0.90	0.91	0.07	0.06 (0.05∼0.07)

TLI = Turker-lewis index; CFI = comparative fit index; SRMR = standardized root-mean-square residual; RMSEA = root mean square error of approximation; CI = confidence interval

**Fig. 3:**
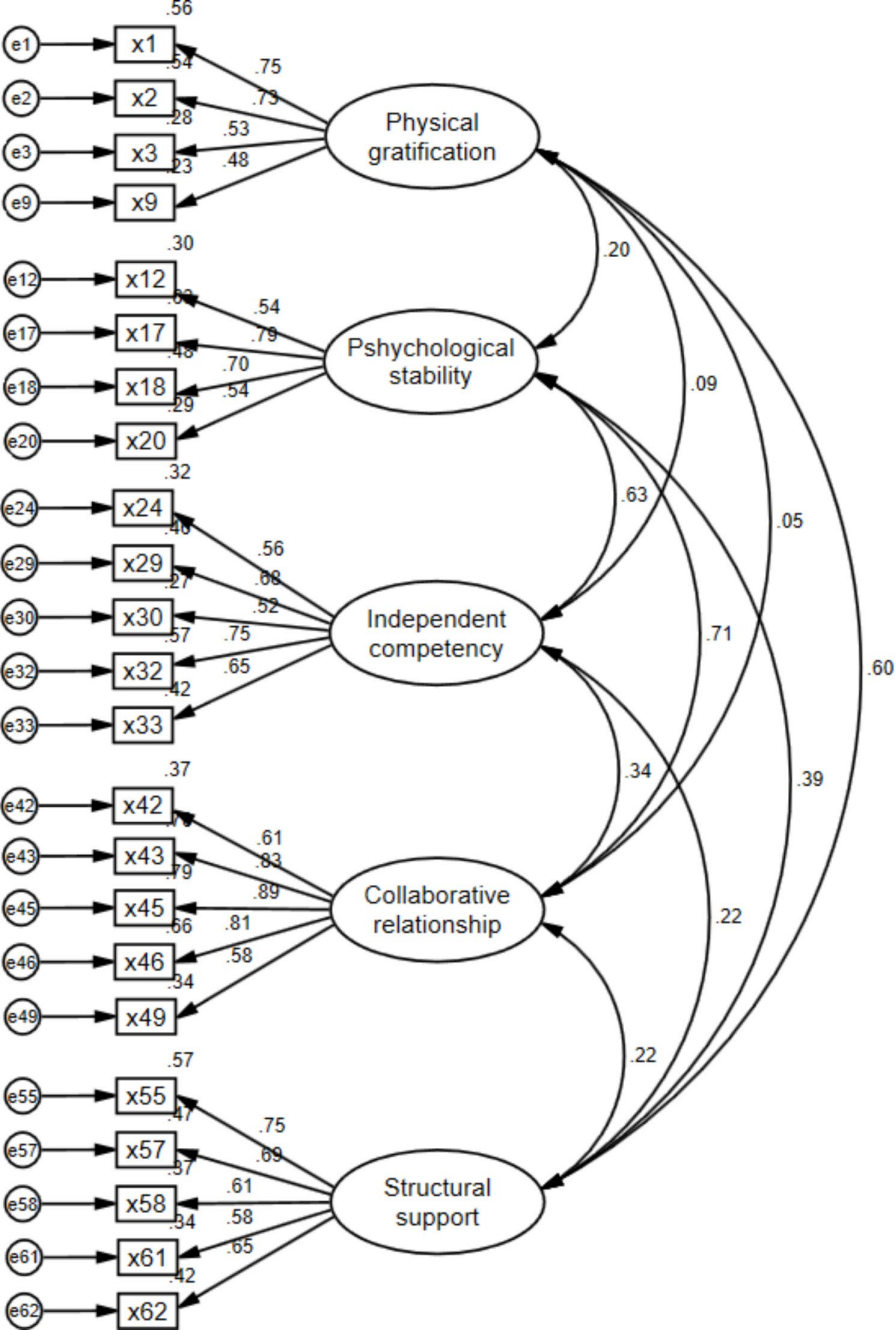
Confirmatory factor analysis results of the research model

The standardized path coefficients (β) for each sub-factor ranged from 0.48 to 0.89, as shown in Fig. 3. The path coefficients and critical ratios for each item are presented in Table 3.

**Table 3:** Confirmatory factor analysis for quality of healthy work environment (N = 247)

Factors	Items	B	SE	β	CR	*p*
Physical gratification	x1. Nurses are guaranteed meal breaks during their shifts.	1.00		0.75		
	x2. Nurses can afford to drink water (beverages) during their shifts.	0.99	0.11	0.73	8.87	< 0.001
	x3. Nurses have time to address menstrual issues during their shifts.	0.67	0.10	0.53	6.86	< 0.001
	x9. Nurses have free access to rest areas during their shifts.	0.67	0.11	0.48	6.21	< 0.001
Psychological stability	x12. Nurses are recognized for their work by their peers/senior nurses.	1.00		0.54		
	x17. Nurses feel a sense of belonging as part of the unit.	1.72	0.22	0.79	7.94	< 0.001
	x18. Nurses feel fulfilled by their nursing work.	1.53	0.20	0.70	7.49	< 0.001
	x20. Nurses feel rewarded while practicing nursing.	1.15	0.18	0.54	6.36	< 0.001
Independent competency	x24. Nurses perform nursing behaviors independently to care for patients.	0.80		0.56		
	x29. Nurses perform mature communication commensurate with their years of experience.	1.00	0.11	0.68	7.41	< 0.001
	x30. Nurses can complete their work within their shift.	1.05	0.15	0.52	6.90	< 0.001
	x32. Nurses do their part in nursing.	1.10	0.12	0.75	9.23	< 0.001
	x33. Nurses experience growth in their job competencies as they progress through their careers.	1.07	0.13	0.65	8.37	< 0.001
Collaborative relationship	x42. Senior nurses in my hospital (department) focus on education rather than blame for mistakes.	1.00		0.61		
	x43. Nurses in my hospital (department) work well as a team.	1.31	0.13	0.83	10.07	< 0.001
	x45. Nurses in my hospital (department) volunteer to help in the interest of teamwork.	1.47	0.14	0.89	10.43	< 0.001
	x46. Nurses in my hospital (department) solve difficult tasks through collaboration.	1.24	0.13	0.81	9.91	< 0.001
	x49. Nurse manager respects the decision-making of the nurses.	1.02	0.13	0.58	7.77	< 0.001
Structural support	x55. Our hospital (department) has a sufficient number of nurses to provide quality care.	1.00		0.75		
	x57. Our hospital (department) assigns patients with severity levels that match the nurses’ competencies.	0.89	0.10	0.69	9.60	< 0.001
	x58. Our hospital (department) has enough time for new nurses to adjust to the job.	0.80	0.10	0.60	8.62	< 0.001
	x61. Our hospital (department) supports professional counseling services when the nurse needs help.	0.71	0.09	0.58	8.19	< 0.001
	x62. Our hospital (department) provides reasonable allowances in addition salary (overtime, night shift, hazardous duty, etc.).	0.94	0.10	0.65	9.07	< 0.001

CR = critical ratio; SE = standard error

Convergent validity and discriminant validity of the sub-factors were confirmed. The AVE values, which are the average of the squared values of the standardized factor loadings, fell below the recommended standard of 0.50 for four factors except social relationships, but the CR values calculated from the standardized factor loadings and error variance values were above the conservative standard of 0.70 for all five sub-factors, thus securing convergent validity. The squared values of the correlation coefficients between the sub-factors were all smaller than the AVE values, confirming discriminant validity (Table 4).

**Table 4:** Convergent and discriminant validity (N = 247)

Factors	CR	AVE	MSV	Inter-concept correlation r (r^2^)			
				Factor 1	Factor 2	Factor 3	Factor 4
Factor 1	0.72	0.40	0.36				
Factor 2	0.74	0.43	0.42	0.20(0.04)			
Factor 3	0.77	0.41	0.41	0.09(0.01)	0.63(0.40)		
Factor 4	0.87	0.57	0.51	0.05(0.01)	0.71(0.51)	0.34(0.12)	
Factor 5	0.79	0.43	0.43	0.60(0.36)	0.39(0.15)	0.22(0.05)	0.22(0.05)

CR = construct reliability; AVE = average variance extract; MSV = maximum shared squared variance; Factor 1 = physical gratification; Factor 2 = psychological stability; Factor 3 = independent competency; Factor 4 = collaborative relationship; Factor 5 = structural support

### Evaluate criterion validity

The correlation coefficient between the K-PES-NWI and the Quality of Healthy Work Environment (QHWE) instrument developed in this study was 0.54 (*p* < .001), indicating a statistically significant positive correlation. When examining the correlations by subscale, the correlation coefficient of factor 1 PES-MWI was 0.28 (*p* < .001), factor 2 and PES-MWI was 0.35 (*p* < .001), factor 3 and PES-MWI was 0.11 (*p* = .081), factor 4 and PES-MWI was 0.44 (*p* < .001), and factor 5 and PES-MWI was 0.51 (*p* < .001), which were statistically significant except for factor 3 (Table 5).

**Table 5:** Correlation between quality of healthy work environment and K-PES-NWI (N = 247)

	QHWE	Factor 1	Factor 2	Factor 3	Factor 4	Factor 5	M ± SD	Skewness	Kurtosis
QHWE							3.18 ± 0.42	− 0.04	− 0.19
Factor 1	0.56(< 0.001)						2.45 ± 0.73	0.25	0.05
Factor 2	0.74(< 0.001)	0.15( 0.022)					3.43 ± 0.58	− 0.24	0.13
Factor 3	0.62(< 0.001)	0.13( 0.046)	0.55(< 0.001)				3.54 ± 0.54	− 0.11	− 0.15
Factor 4	0.61(< 0.001)	0.03( 0.605)	0.55(< 0.001)	0.28(< 0.001)			3.73 ± 0.59	− 0.02	0.01
Factor 5	0.73(< 0.001)	0.44(< 0.001)	0.32(< 0.001)	0.21( 0.001)	0.19( 0.003)		2.66 ± 0.77	− 0.03	− 0.25
K-PES-NWI	0.54(< 0.001)	0.28(< 0.001)	0.35(< 0.001)	0.11( 0.081)	0.44(< 0.001)	0.51(< 0.001)	2.64 ± 0.38	0.02	0.97
PES-P	0.42(< 0.001)	0.24(< 0.001)	0.27(< 0.001)	0.02( 0.796)	0.36(< 0.001)	0.43(< 0.001)	2.53 ± 0.48	− 0.08	0.31
PES-F	0.50(< 0.001)	0.20( 0.002)	0.35(< 0.001)	0.19( 0.003)	0.39(< 0.001)	0.46(< 0.001)	2.88 ± 0.39	− 0.04	0.85
PES-M	0.38(< 0.001)	0.11( 0.086)	0.33(< 0.001)	0.02( 0.709)	0.54(< 0.001)	0.27(< 0.001)	2.86 ± 0.49	− 0.41	0.75
PES-S	0.53(< 0.001)	0.44(< 0.001)	0.22( 0.001)	0.12( 0.057)	0.23(< 0.001)	0.59(< 0.001)	2.12 ± 0.55	0.26	0.32
PES-R	0.33(< 0.001)	0.15( 0.019)	0.21( 0.001)	0.19( 0.003)	0.31(< 0.001)	0.23(< 0.001)	2.66 ± 0.53	− 0.33	0.58

QHWE = Quality of Healthy Work Environment Instrument; Factor 1 = physical gratification; Factor 2 = psychological stability; Factor 3 = independent competency; Factor 4 = collaborative relationship; Factor 5 = structural support K-PES-NWI = Korean Practice Environment Scale of the Nursing Work Index; PES-*P* = nurse participation in hospital affairs; PES-F = nursing foundations for quality of care; PES-M = nurse manager ability, leadership, and support of nurses; PES-S = staffing and resource adequacy; PES-R = collegial nurse–physician relations

### Evaluate reliability

The overall Cronbach’s ⍺ coefficient of the developed in this study was 0.85. By sub-factor, it was 0.70 for factor 1, 0.76 for factor 2, 0.75 for factor 3, 0.86 for factor 4, and 0.79 for factor 5. In addition, when the test-retest reliability was analyzed, the correlation coefficient between the first and second surveys was 0.73 (*p* < .001), which ensured the stability of the instrument. The correlation coefficient of test-retest reliability by sub-factor was 0.69 (*p* < .001) for factor 1, 0.60 (*p* < .001) for factor 2, 0.48 (*p* = .006) for factor 3, 0.61 (*p* < .001) for factor 4, and 0.66 (*p* < .001) for factor 5.

### Optimization of instrument

The QHWE instrument for shift nurses was finalized to include 23 items with five components. The items were scored on a Likert scale ranging from 1 “strongly disagree” to 5 “strongly agree”, and the distribution of scores ranged from a low of 23 to a high of 115. The five components identified were named as follows based on a conceptual framework that implies the content of the items: (1) physical gratification (4 items), (2) psychological stability (4 items), (3) independent competency (5 items), (4) collaborative relationship (5 items), and (5) structural support (5 items). When the total score was calculated, the higher the score, the higher the quality of healthy work environment perceived by nurses.

## Discussion

This study derived a conceptual framework based on WHO’s health-related quality of life attributes and AACN’s standards for a healthy nursing work environment, and developed preliminary items to measure the quality of a healthy work environment through a literature review and interviews with shift nurses. The validity of the instrument was evaluated through confirmatory factor analysis with shift nurses.

In the final set of items adopted for this study, the first component, “physical gratification”, consisted of four items related to meal breaks, time to quench thirst and physiological needs, and free access to rest areas. The results confirmed that nurses’ physical comfort was maintained by ensuring meal breaks, satisfying physiological needs, providing resting areas, and when the intensity of work was appropriately guaranteed to prevent physical strain, which are factors that determine the quality of a healthy work environment. Previous studies have also found that a physical factor contributing to the poor quality of nurses’ work environment is the level of fatigue experienced by nurses and that increased fatigue is associated with decreased nursing performance [42]. In addition, unmet physiological needs increased turnover intentions among shift nurses [43, 44]. Previous studies on nurses’ work environments and AACN’s healthy nursing work environment standards did not directly assess physical factors [12, 13, 15, 23]. This study addresses this gap by developing a questionnaire to measure factors influencing physical health. As nurses are required to work three shifts because of the nature of their work, which requires them to be at the patient’s bedside 24 hours a day, they should not ignore factors in their work environment that affect their physical health. In a survey of nurses’ human rights violations in Korea, 31.1% of nurses did not have guaranteed meal breaks and 54.4% of nurses did not have guaranteed rest breaks and reported working conditions that were detrimental to their physical health [45]. In addition, a qualitative study of shift nurses, they reported that they longed for the freedom to take meal breaks during work and were worried about changing jobs because of concerns about their physical health [43]. Breaks are defined as uninterrupted periods of at least 20 min to recover from physical and mental strain, and no work should be performed during these periods [46]. In the United States, state labor laws require a minimum of 30 minutes for meal breaks [47]. Several studies have reported nurses’ tendency to miss breaks or stop eating during the workday [46, 48] and emphasized the need for breaks during work [49]. These poor working conditions lead to nurses leaving the workplace, which in turn leads to nursing shortages and further aggravates the working conditions, resulting in a vicious cycle. Therefore, to improve the quality of a healthy work environment for nurses, it is important to create a work environment that satisfies their basic physiological needs and promotes physical safety and comfort.

The second component “psychological stability” consisted of four items related to the positive emotions experienced by shift nurses while working in the field. The results confirmed that nurses’ perceptions of being recognized by their peers, feeling a sense of belonging, and being rewarded were factors affecting healthy work environment. The AACN’s standards for a healthy nursing work environment define a healthy workplace as one in which nurses receive appropriate recognition for their work and growth under the ‘meaningful recognition’ factor [23], the results of this study are similar to those of this study. Nurses found recognition from patients, their families, and fellow nurses in the team to be most meaningful, and such recognition was positively associated with increased job satisfaction, decreased turnover intentions, and quality of care [17, 50]. In this study, the sense of accomplishment and reward experienced by nurses, not only through their work but also through team and individual recognition, is a factor that promotes their mental health. This is the most unique factor that distinguishes this study from previous studies related to the nursing work environment. Previous studies comprised items about positive experiences through the personal behavior of nursing managers [13]. Another study asked about nurse managers’ ability to support nurses in decision-making and provide praise and recognition [12], which are categorized as the domain of nurse managers [12, 13]. In this study, we took a difference by confirming that the quality of the healthy work environment can also depend on the personal experiences of nurses, not nurse managers. In other words, the more positive emotional experiences nurses have through their work, the better the quality of a healthy work environment. Therefore, it is necessary for nurses to develop their competencies and experience fulfillment and reward through their work. Additionally, it is necessary to create an organizational culture in which all members, including nursing managers, provide meaningful recognition and encouragement to each other to increase positive emotional experiences.

The third component “independent competency” consisted of five items related to nurses’ independence and scope of work. The study found that nurses’ capability to complete their assigned tasks on time and do their share of the work, and to experience growth in their nursing competencies over the course of their careers were factors affecting the quality of a healthy work environment. The AACN’s healthy work environment standards for nurses identify their participation in the decision-making process of patient care and influence the quality of care as a component of healthy working environment under the factor of “effective decision-making” [23]. The domestically developed nursing work environment measurement tool comprised items that measured nurses’ independent work performance and clarification of work scope [13] that was an active reflection of nurses’ work competence in professional practice areas [15]. There were items that measured nurses’ independent competence. This study partially agrees with the previous studies by focusing on the scope and competence of nurses’ independent work to measure the quality of their healthy work environment. However, independent competence was not significantly correlated with “nurse participation in hospital affairs”, “nurse manager ability, leadership, and support of nurses”, and “staffing and resource adequacy” among the sub-factors of the K-PES-NWI used as validity measures. This suggests that the independent competency items developed in this study are unique items that can assess the quality of a healthy work environment, which has not been assessed by existing measurement instruments. An environment in which nurses can actively practice nursing and experience growth as they progress through their careers is critical to their profession. Nurses’ professional autonomy of nurses means that they are trained based on their professional knowledge and sense of responsibility and perform their nursing duties by making reasonable decisions through good communication [51]. In other words, nurses’ professionalism can be exerted in an environment in which their independent competence and growth are guaranteed. Additionally, previous studies have shown that professional autonomy predicts nurses’ job stress and increases job satisfaction [52, 53]. Ensuring nurses’ autonomy through independent competencies should be considered in order to promote healthy work environment for nurses. Previous studies have shown that nurses’ professional autonomy has a significant effect on job satisfaction [53, 54]. Therefore, establishing clear roles and professional autonomy for nurses are important key indicators of a healthy work environment. To improve the quality of healthy working environment for nurses, there should be continuous support for them to clarify their scope of work and grow their work capacity to ensure independent autonomy in nursing field, including medical institutions.

The fourth component “collaborative relationship” consisted of five items related to the teamwork and the true collaboration that nurses experience on the job. In this study, nurses identified working in a teamwork environment, volunteering to help in teamwork, collaboratively solving difficult tasks, and being respected by nurse managers as factors affecting the quality of a healthy work environment. Among the sub-factors of the K-PES-NWI instrument utilized as a quasi-validity measure, nurse manager ability, leadership, and support of nurse’ showed a high correlation of more than 0.50. In nursing practice, the concept of collaboration is “an intra- or interprofessional process in which team members respectfully share knowledge and resources to solve patient care or healthcare system problem [55]”. The “cooperative relationship component” in this study faithfully reflects this concept of “collaboration”. The evaluation of essential magnetism (EOM), a magnet certification measure for nurse retention in the United States, emphasizes that a healthy work environment is interpersonally oriented [25]. In addition, AACN’s healthy working environment standards suggest pursuing “true collaboration” and having a structured process for such collaboration [23]. This study also found that interpersonal relationships and teamwork among nurses were determinants of the quality of a healthy work environment, confirming previous research that teamwork and collaboration are fundamental factors in achieving patient care [23, 25, 32]. On the other hand, while the domestically developed nursing work environment measurement instruments focus on the relationship and cooperation with physicians and the leadership of nursing managers [12, 13], this study differed from previous studies by selecting one item, “The nursing manager respects the decision-making of the nurse in charge,” as a question about nursing managers. Interprofessional teamwork and cooperation have been identified as effective strategies for healthcare delivery in primary healthcare organizations [56]. In addition, nurse-physician collaboration and nurse-nurse collaboration have been found to be significantly associated with patient safety outcomes in acute care settings [5, 57]. Effective communication and collaboration have also been shown to increase nurses’ job satisfaction, quality of care, and decrease turnover intentions [17]. Thus, teamwork and collaboration among nurses are crucial for patient safety and providing the best care. To improve the quality of a healthy work environment, regular training and practice must be conducted for nurses to develop their skilled communication skills, and efforts should be made to improve the atmosphere of nursing organizations to foster collaborative teamwork.

The fifth component “structural support” consisted of five items related to the systemic support provided by the healthcare organization. This study found that an adequate number of nurses, assignment of patients according to severity, educational support for new nurses, support for professional counseling services, and reasonable allowances were factors affecting the quality of a healthy work environment. Among the sub-factors of the K-PES-NWI instrument utilized as a quasi-validity measure, “staffing and resource adequacy” showed a high correlation of more than 0.50. These results are consistent with previous research on administrative and financial support of healthcare organizations, such as “supportive policies“ [13] and “sufficient personnel and material support“ [12]. Previous studies have also shown that sufficient staffing is necessary to create a desirable nursing work environment [12, 13] and the need to provide physical resources [12]. In addition, it was emphasized that staffing that considers patient severity and nurse competence is an essential factor in the nursing work environment [15]. In a study of the relationship between staffing, work environment, and readmission of surgical patients in the United States, the quality of nurses’ work environment and adequacy of staffing were significantly associated with readmission [58]. The AACN’s healthy nursing working environment standards also identify adequate staffing as a key component [23]. To address work intensity that impairs nurses’ health, organizations should strive to ensure adequate staffing. Insufficient nurse staffing levels and excessive workloads have been strongly associated with nurse burnout, which reduces the quality of care delivered to patients and threatens patient safety [59, 60]. Turnover rates for new nurses continue to rise, with 52.8% of new nurses resigning within one year in 2022 in Korea [61]. Whether new nurses experienced a supportive environment in the first six months determined the success of their adjustment, the more positively new nurses perceived the working environment in the hospital, the lower the transition shock to the new environment [62]. This study also confirmed the importance of providing an appropriate period for new nurses to adapt to their work and professional counseling services when necessary to create a healthy work environment. As such, structural supports, such as ensuring an adequate number of nurses and appropriate patient assignments according to severity, supporting new nurses in their work, accessing professional counseling services, and reasonable allowances, are essential for the quality of nurses’ healthy work environment. To ensure a healthy work environment for nurses, a systematic approach based on an understanding of the complexity of nursing workplaces, and active financial and administrative support should be provided.

To develop an instrument to evaluate the quality of a healthy work environment for shift nurses in Korea, this study refined the questionnaire through the establishment of content validity by experts, interviews with shift nurses, and evaluation of questionnaire understanding through a preliminary survey. The K-PES-NWI was used as a standardized measurement instrument in related fields, and its content validity, construct validity, and high reliability were reported at the time of development. It is significant that the existing nursing work environment instruments cover organizational culture, staffing, communication, collaboration, and leadership of nursing managers, whereas the instrument developed in this study covers aspects of physical, psychological, and independent factors of nurses. Nevertheless, the correlation between the sub-factors was not significant for component 1 (physical gratification) or component 4 (collaborative relationship), limiting its validity. Future research should repeat the study to reconfirm the relevance of physical gratification and collaborative relationships in constructing the categories of the quality of healthy work environment. In addition, there is a limitation that the conceptual framework did not address the “spiritual domain” among the sub-factors of health-related quality of life proposed by the WHO. We recommend that future research extend the conceptual framework by analyzing the “spiritual domain” as a component of a healthy work environment. The scale of the instrument developed in this study was composed of 5 points. This odd-numbered scale tends to cause respondents to set an intermediate evaluation score, so careful interpretation is required. It is necessary to further refine the instrument by applying a 4-point or 6-point response method in subsequent studies. Finally, the generalizability of the findings is limited because of the geographically biased sample. Therefore, we recommend a replication study with cross-validation to evaluate the quality of nurses’ healthy work environment by expanding this study to various regions and medical institutions.

## Conclusions

The QHWE instrument was found to be reliable and valid to quantitatively measure nurses’ perceived quality of healthy work environment. The QHWE consists of 23 items and 5 components. The QHWE is measured on a 5-point Likert scale, and a higher total score is interpreted as a nurse’s perceived healthy work environment. By measuring the components of physical gratification, psychological stability, independent competence, collaborative relationship, and structural support from the perspective of shift nurses, the QHWE can assess the quality of a healthy work environment and provide meaningful information about nursing work. Furthermore, identifying the factors that contribute to health and unhealth in nurses’ work environments, it can be used to reduce job stress and prevent premature turnover. By exploring ways to maintain and promote health through the creation of a desirable nursing work environment, we can contribute to the development of policies to improve the quality of life of shift nurses.

The academic and practical significance of this research can be summarized as follows. The academic contribution is to develop an instrument that can quantitatively measure the quality of a healthy work environment for nurses and to promote research on healthy work environments to improve the health of nurses. Practical Significance is that it can increase awareness and attention to healthy work environments for nurses in clinical settings. In addition, this instrument can contribute to the improvement of a healthy and desirable working environment for nurses and provide a basis for the development of programs to promote a healthy work environment. Since this instrument was developed based on the Korean cultural background, cross-cultural validity testing is required when applying it in other cultures. We look forward to further research on the quality of nurses’ healthy work environment in other cultures.

### Electronic supplementary material

Below is the link to the electronic supplementary material.

Supplementary Material 1

## Data Availability

The dataset supporting the conclusions is available from the corresponding author on reasonable request.
