# Supplementary material for: Development and validation of a quality of healthy work environment instrument for shift nurses
Source: BMC Nurs. 2024 Jan 12;23:37. doi: 10.1186/s12912-023-01672-4 (PMC10785413; doi:10.1186/s12912-023-01672-4)
Supplement: Supplementary file 1 — Supplementary Material 1 [file 12912_2023_1672_MOESM1_ESM.docx]

**In-depth interview questionnaire to develop a**

**"Healthy Workplace Measurement Instrument"**

What do you consider a healthy work environment?

What do you consider an unhealthy work environment?

What do you consider to be a physically fulfilling work environment?

What do you think makes a work environment psychologically (mentally) stable?

What do you think is a work environment that allows you to practice your work capabilities independently?

What do you think fosters social (collaborative) relationships in the workplace?

What do you consider to be a structured (institutional) supportive work environment?

What is the environment in which you work that does not endanger your health (well-being)?

When have you experienced a healthy environment to work in?

What factors do you think improve (enhance) a healthy work environment for nurses?

What factors do you think contribute to endanger a healthy work environment for nurses?
